# Supplementary material for: Taxonomy of Pseudomonas spp. determines interactions with Bacillus subtilis
Source: mSystems. 2024 Sep 10;9(10):e00212-24. doi: 10.1128/msystems.00212-24 (PMC11494997; doi:10.1128/msystems.00212-24)
Supplement: Supplemental figures and table — Fig. S1 and S2; Table S1. [file msystems.00212-24-s0002.pdf]

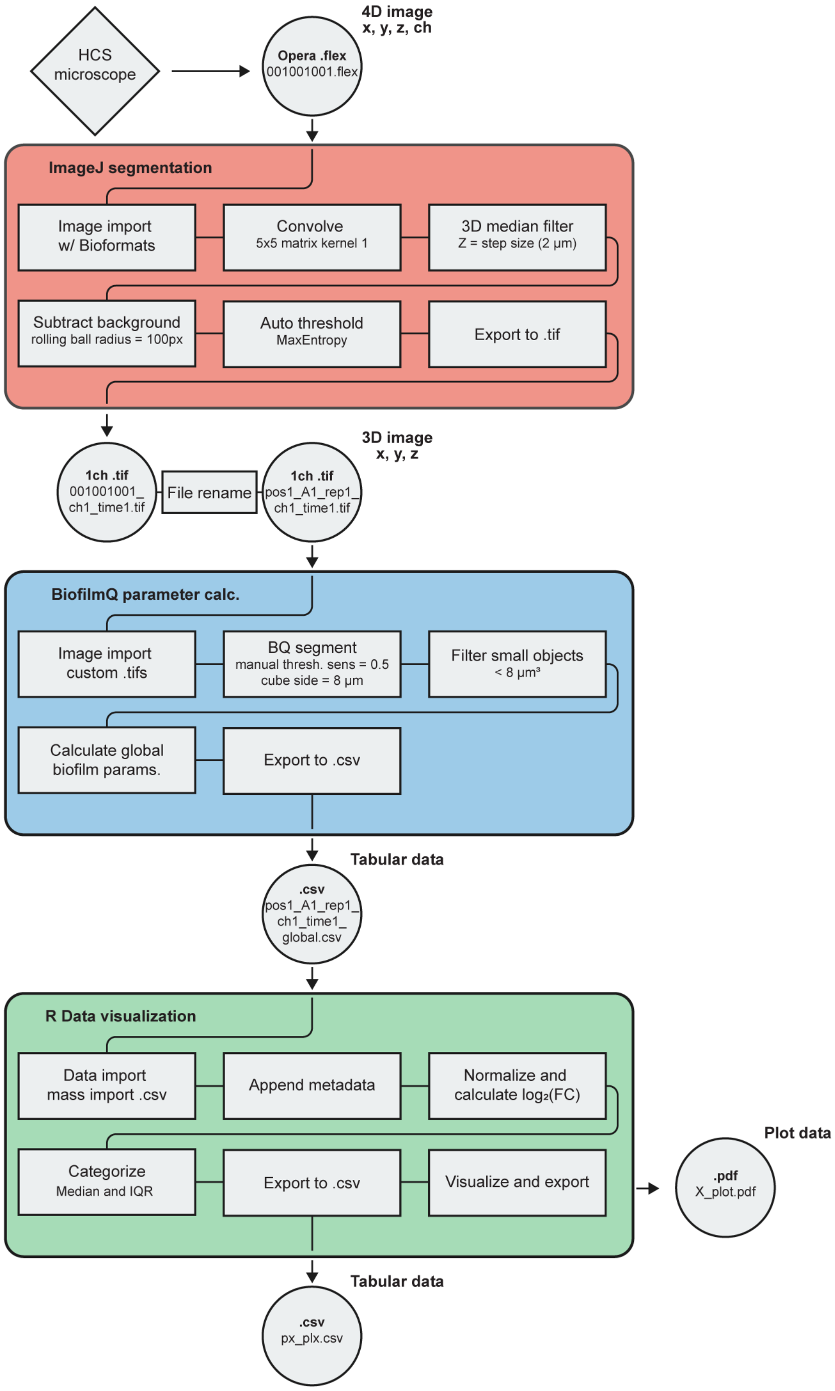

Figure S1 – Image analysis flowchart. Images were acquired with an Opera High Content Screening microscope and processed through a custom image analysis workflow. Preprocessing and segmentation were performed in ImageJ by convolving on a square grid, filtering small objects with a 3D median filter, and subtracting background signal via the built-in rolling ball method. A threshold was applied by the MaxEntropy algorithm, and the binary images were exported as one .tif file per channel. Biofilm parameters were calculated with BiofilmQ on the manually segmented images, applying a cube with sides of length 8 µm as pseudocells. Objects smaller than this were discarded, and global biofilm parameters (e.g., biovolume) were written to a comma-separated file. The tabular data was then read into R for further data analysis.

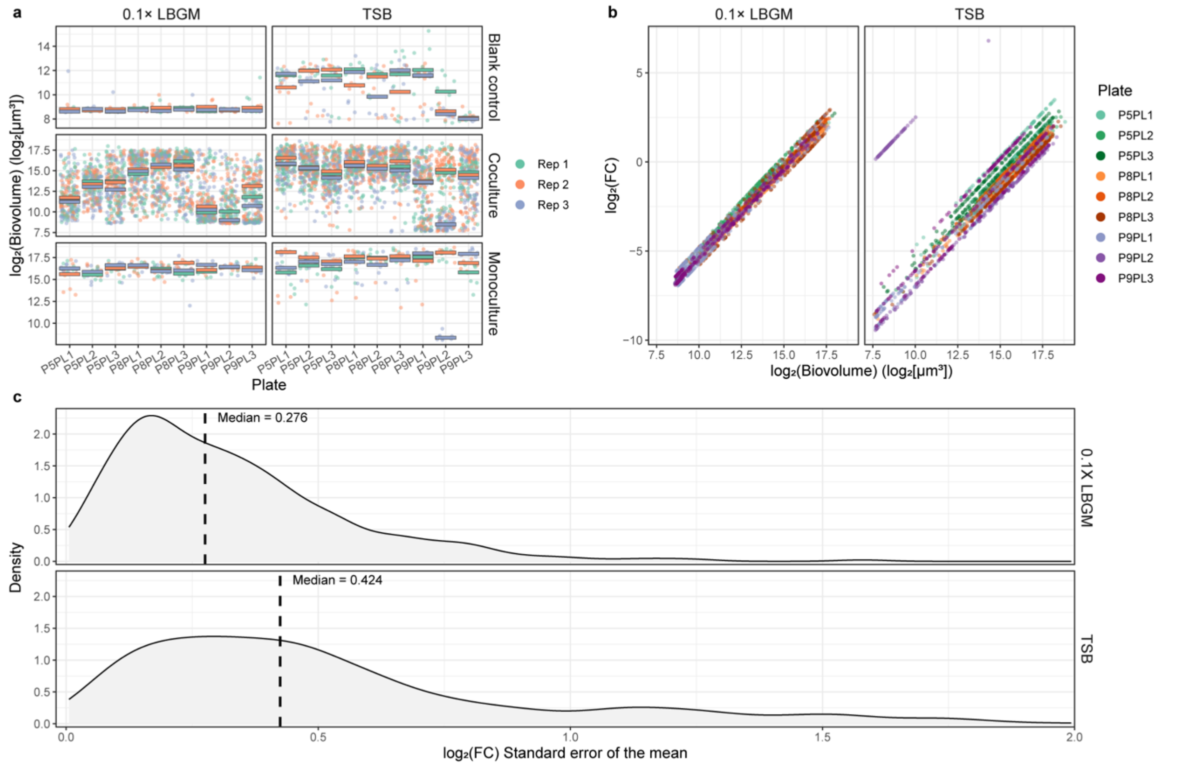

Figure S2 – Biofilm biovolume variance. a) log-transformed biofilm biovolume of DK1042 in two types of media (columns) for three sample types (rows). Points represent individual data points colored by replicate. Horizontal bars represent medians of all samples. The X-axis represents 96-well library plates. P5, P8, and P9 denote the isolation site, and PL1, 2, and 3 denote the plate number. The medians are expected to be approximately equivalent across replicates. The TSB blank controls generally varied more than 0.1×LBGM, which may have skewed log2FC calculations. Cocultures with pseudomonads from P9 generally were more often negative than those from other isolation sites (in 0.1×LBGM). **b)** log2FC as a function of log2(biovolume). As log2FC is calculated from the biovolume, these are expected to be perfectly correlated. The difference in y-intercept between plates, reveals a higher variance in cocultures cultivated in TSB compared with 0.1×LBGM. **c)** Distribution of standard error of the mean for log2FCs calculated from cocultures in the two media types. The wider distribution and higher median of the TSB values, corroborates the finding in b).

Dataset S1 – Summarized biofilm biovolumes. This dataset reports the median biovolume and median log2FC for blank controls, DK1042 grown in monoculture, or DK1042 grown in coculture with each of the 719 soil isolates. Columns denote the library plate, the growth medium (0.1×LBGM or TSB), the culture type (blank, mono- or coculture), the median biovolume [um^3], the median log2FC, the log2FC first quartile, the log2FC third quartile, the log2FC interquartile range, the log2FC standard error of the mean, and the designated category of the isolate. This data can be used to remake figures 1 and S2.

Table S1 – Strains and oligos

Strain

Bacillus subtilis	**Genotype**		**Ref**
B. subtilis DK1042	3610 $comI^{Q12I}$		[1]
TB501.1	3610 $comI^{Q12I}$ *amyE*::$P_{hyperspank}$-mKate2-$Spec^{R}$		[2]
TB864	3610 $comI^{Q12I}$ *amyE*::$P_{hyperspank}$-mKate2-*cat*, P_{eps}-eGFP-Km^{R}		[2]

Pseudomonas	**Genotype**	**Genome Accession**	**Ref**
Pseudomonas sp. P5_109	WT isolate	CP125380	[3]
Pseudomonas sp. P5_152	WT isolate	JASFAH000000000	[3]
P. zeae P8_72	WT isolate	JASFAG000000000	[3]
Pseudomonas sp. P8_139	WT isolate	CP125379	[3]
Pseudomonas sp. P8_229	WT isolate	CP125378	[3]
Pseudomonas sp. P8_241	WT isolate	CP125377	[3]
Pseudomonas sp. P8_250	WT isolate	JASFAF000000000	[3]
Pseudomonas sp. P9_31	WT isolate	CP125375	[3]
Pseudomonas sp. P9_2	WT isolate	CP125376	[3]
Pseudomonas sp. P9_32	WT isolate	CP125374	[3]
Pseudomonas sp. P9_35	WT isolate	CP125373	[3]
P. germanicum P9_87	WT isolate	CP125372	[3]
P. protegens P9_191	WT isolate	JASFAE000000000	[3]
P. lini 1.6	WT isolate		Unpublished
P. poae DSM 14936	Type strain	LT629706	[4]
P. kermanshensis F8	WT isolate	CP099575	[5]
P. protegens DTU9.1	WT isolate	CP024025	[5]

Escherichia coli	**Genotype**		**Ref**
E. coli CC118	CC118 λpir/pBG42		[5]
E. coli HB101	HB101 /pRK600		[5]
E. coli CC118	CC118 λpir/pTNS2		[5]

**Oligo ID**	**Description**	**Sequence (5' – 3')**
PsEG30F-BC13	Positive 1.1 – Fw	ATTGCTGAATYGAAATCGCCAARCG
PsEG30F-BC14	Positive 1.2 – Fw	TGAGTTCTATYGAAATCGCCAARCG
PsEG30F-BC15	Positive 2.1 – Fw	GGCTATTTATYGAAATCGCCAARCG
PsEG30F-BC16	Positive 2.2 – Fw	CAAGAGATATYGAAATCGCCAARCG
PsEG30F-BC17	Negative 1.1 – Fw	GGAATACAATYGAAATCGCCAARCG
PsEG30F-BC18	Negative 1.2 – Fw	AAGGCAATATYGAAATCGCCAARCG
PsEG30F-BC19	Negative 2.1 – Fw	ACAAAACGATYGAAATCGCCAARCG
PsEG30F-BC20	Negative 2.2 – Fw	TTGAGTGAATYGAAATCGCCAARCG
PsEG30F-BC21	Neutral 1.1 – Fw	GCTTCTGAATYGAAATCGCCAARCG
PsEG30F-BC22	Neutral 1.2 – Fw	GGCAAGATATYGAAATCGCCAARCG
PsEG30F-BC23	Neutral 2.1 – Fw	GTGCTTTCATYGAAATCGCCAARCG
PsEG30F-BC24	Neutral 2.2 – Fw	ACACACTGATYGAAATCGCCAARCG
PsEG30F-BC25	Total 1.1 – Fw	CGATTCTGATYGAAATCGCCAARCG
PsEG30F-BC26	Total 1.2 – Fw	GCAGAGTTATYGAAATCGCCAARCG
PsEG30F-BC27	Total 2.1 – Fw	CGTCCTATATYGAAATCGCCAARCG
PsEG30F-BC28	Total 2.2 – Fw	GCTTGGTTATYGAAATCGCCAARCG
PsEG30F-BC29	Empty control 1 – Fw	ACAGGCTTATYGAAATCGCCAARCG
PsEG30F-BC30	Empty control 1 – Fw	TGACGCTTATYGAAATCGCCAARCG
PsEG790R-BC13	Positive 1.1 – Rv	ATTGCTGACGGTTGATKTCCTTGA
PsEG790R-BC14	Positive 1.2 – Rv	TGAGTTCTCGGTTGATKTCCTTGA
PsEG790R-BC15	Positive 2.1 – Rv	GGCTATTTCGGTTGATKTCCTTGA
PsEG790R-BC16	Positive 2.2 – Rv	CAAGAGATCGGTTGATKTCCTTGA
PsEG790R-BC17	Negative 1.1 – Rv	GGAATACACGGTTGATKTCCTTGA
PsEG790R-BC18	Negative 1.2 – Rv	AAGGCAATCGGTTGATKTCCTTGA
PsEG790R-BC19	Negative 2.1 – Rv	ACAAAACGCGGTTGATKTCCTTGA

PsEG790R-BC20	Negative 2.2 – Rv	TTGAGTGACGGTTGATKTCCTTGA
PsEG790R-BC21	Neutral 1.1 – Rv	GCTTCTGACGGTTGATKTCCTTGA
PsEG790R-BC22	Neutral 1.2 – Rv	GGCAAGATCGGTTGATKTCCTTGA
PsEG790R-BC23	Neutral 2.1 – Rv	GTGCTTTCCGGTTGATKTCCTTGA
PsEG790R-BC24	Neutral 2.2 – Rv	ACACACTGCGGTTGATKTCCTTGA
PsEG790R-BC25	Total 1.1 – Rv	CGATTCTGCGGTTGATKTCCTTGA
PsEG790R-BC26	Total 1.2 – Rv	GCAGAGTTCGGTTGATKTCCTTGA
PsEG790R-BC27	Total 2.1 – Rv	CGTCCTATCGGTTGATKTCCTTGA
PsEG790R-BC28	Total 2.2 – Rv	GCTTGGTTCGGTTGATKTCCTTGA
PsEG790R-BC29	Empty control 1 – Rv	ACAGGCTTCGGTTGATKTCCTTGA
PsEG790R-BC30	Empty control 1 – Rv	TGACGCTTCGGTTGATKTCCTTGA
B2BF	*phlD* – Fw	ACCCACCGCAGCATCGTTTATGAGC
BPR4	*phlD* – Rv	CCGCCGGTATGGAAGATGAAAAAGTC
PsEG30F	*rpoD* – Fw	ATYGAAATCGCCAARCG
PsEG790R	*rpoD* – Rv	CGGTTGATKTCCTTGA

Underlined: Barcode for amplicon sequencing.
PsEG30F, PsEG790R, and their barcoded derivatives are from [6].

[1] Konkol MA, Blair KM, Kearns DB (2013) Plasmid-encoded ComI inhibits competence in the ancestral 3610 strain of *Bacillus subtilis*. *J Bacteriol* 195:4085-4093.
[2] Dragoš A, Kiesewalter H, Martin M, Hsu CY, Hartmann R, et al (2018) Division of labor during biofilm matrix production. *Curr Biol* 28:1903-1913.
[3] Lyng M, Jørgensen JPB, Schostag MD, Jarmusch SA, Aguilar DKC, et al (2024) Competition for iron shapes metabolic antagonism between *Bacillus subtilis* and *Pseudomonas marginalis*. *ISME J* 18:wrad001.
[4] Behrendt U, Ulrich A, Schumann P (2003) Fluorescent pseudomonads associated with the phyllosphere of grasses; *Pseudomonas trivialis* sp. nov., *Pseudomonas poae* sp. nov. and *Pseudomonas congelans* sp. nov *Int J Syst Evol Microbiol* 53:1461-1469.
[5] Hansen ML, Wibowo M, Jarmusch SA, Larsen TO, Jelsbak L (2022) Sequential interspecies interactions affect production of antimicrobial secondary metabolites in Pseudomonas protegens DTU9.1. *ISME J* 16:2680-2690.
[6] Mulet M, Bennasar A, Lalucat J, García-Valdés E (2009) An *rpoD*-based PCR procedure for the identification of *Pseudomonas* species and for their detection in environmental samples. *Mol Cell Probes* 23:140-147.
